# Quantifying Nucleoporin Stoichiometry Inside Single Nuclear Pore Complexes In vivo

**DOI:** 10.1038/srep09372

**Published:** 2015-03-23

**Authors:** Lan Mi, Alexander Goryaynov, Andre Lindquist, Michael Rexach, Weidong Yang

**Affiliations:** 1 Department of Biology, Temple University, Philadelphia, PA 19122; 2 Department of Molecular, Cell and Development Biology, University of California, Santa Cruz, CA 95064; 3 Department of Optical Science and Engineering, Shanghai Engineering Research Center of Ultra-Precision Optical Manufacturing, Fudan University, Shanghai 200433, China

## Abstract

The nuclear pore complex (NPC) is one of the largest supramolecular structures in eukaryotic cells. Its octagonal ring-scaffold perforates the nuclear envelope and features a unique molecular machinery that regulates nucleocytoplasmic transport. NPCs are composed of ~30 different nucleoporins (Nups), averaged at 8, 16 or 32 copies per NPC. This estimate has not been confirmed for individual NPCs in living cells due to the inherent difficulty of counting proteins inside single supramolecular complexes. Here we used single-molecule SPEED microscopy to directly count the copy-number of twenty-four different Nups within individual NPCs of live yeast, and found agreement as well as significant deviation from previous estimates. As expected, we counted 8 copies of four peripheral Nups and 16 copies of fourteen scaffold Nups. Unexpectedly, we counted a maximum of 16 copies of Nsp1 and Nic96, rather than 32 as previously estimated; and found only 10–15 copies of six other Nups, rather than 8 or 16 copies as expected. This *in situ* molecular-counting technology can test structure-function models of NPCs and other supramolecular structures in cells.

Nuclear pore complexes (NPCs) embedded in the nuclear envelope (NE) gate the exchange of macromolecules between the nucleus and the cytoplasm of cells. Each NPC is a large assembly of multiple copies of approximately thirty different proteins termed nucleoporins (Nups)^1^,^2^. At ~45–60 MDa in mass, it represents one of the largest and most complicated machines in eukaryotic cells. Electron microscopic reconstructions have revealed its overall shape. In yeast and vertebrates, it features a canonical ring-spoke scaffold structure with eight-fold rotational symmetry, a central transporter/plug structure, and peripheral fibers protruding into the cytoplasm and nucleoplasm^3^,^4^,^5^,^6^.

Resolving the molecular architecture of NPCs has been a formidable challenge. Besides high resolution cryo-electron micrographs of NPCs^7^,^8^,^9^, computational methods have been used to predict 3D-architecture maps of the yeast NPC^10^,^11^,^12^. By design however the *in silico* modeling required user input of estimated subunit stoichiometries. The stoichiometry of Nups within the yeast and vertebrate NPCs has been majorly estimated by gel densitometry of isolated NPCs^1^,^2^,^11^. Also, the fluorescence emitted by GFP-tagged Nups within yeast was used to estimate relative Nup abundances at NPCs^13^, but the fluorescence signals detected by flow cytometry included all GFP-Nup molecules in the cell, not only those at NPCs. A similar, more sophisticated measurement was conducted on rat kidney cells using confocal microscopy analysis of NPCs labeled with GFP-Nups^14^. Lastly, super-resolution microscopy of isolated NPCs was used to fit a given number of scaffold Nups within current models of average NPCs^15^,^16^. In the end however, due to the inherent difficulty of counting proteins within individual supramolecular complexes in live cells, all of the above reports of Nup stoichiometry per NPC represent best estimates of the average, rather than direct, eye-witness counts from individual NPCs.

A more accurate account of Nup copy-number within individual NPCs of live cells should produce better and more realistic 3D-architecture map of NPCs. Precise information on subunit stoichiometry could change our understanding of the function, biogenesis and compositional dynamics of NPCs. Here, we utilized a single-molecule fluorescence imaging approach called single-point edge-excitation sub-diffraction (SPEED) microscopy^17^,^18^ to directly count the copy-number of GFP-Nup molecules within individual NPCs of live yeast. This novel approach permitted a real-time visualization of Nups within single NPCs, and their quantitation produced a significantly-revised report of subunit stoichiometry for the *S. cerevisiae* NPC.

## Results

### Counting GFP molecules in GFP oligomers

**Figure 1:**
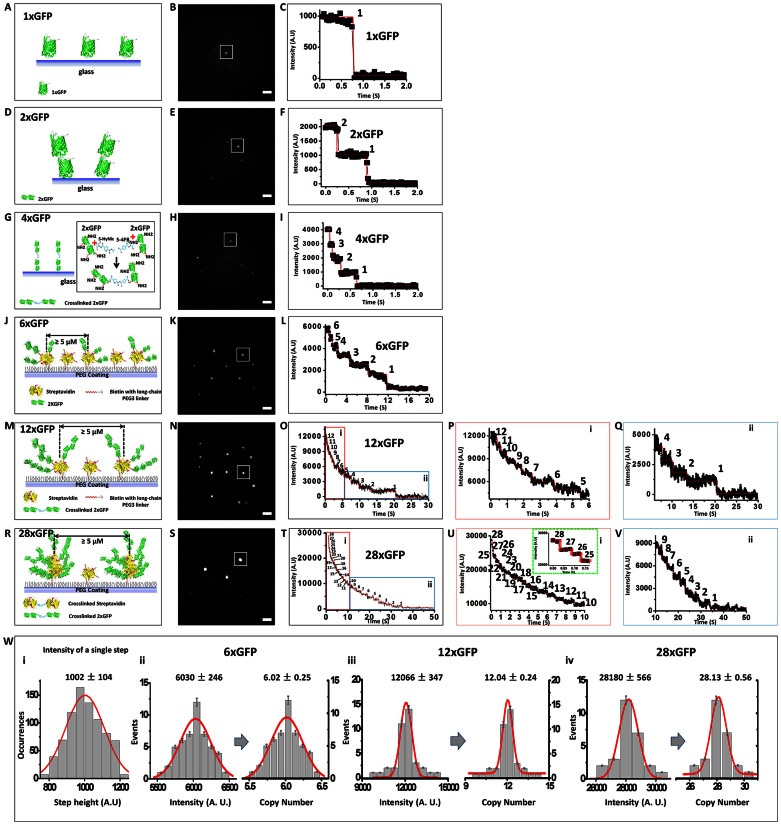
Counting the number of GFP molecules in synthetic protein complexes using SPEED microscopy. (A) Diagram shows 1xGFP anchored on the surface of coverslip. (B) Wide-field epifluorescence images of 1xGFP. The fluorescent spot in the square was analyzed by SPEED microscopy; the spot generated the photobleaching curve shown in C. (C) The photobleaching curve of a 1xGFP molecule. The black dots represent the fluorescent signal; the red line is the fitted photobleaching steps; the number is the step count. (D–F) The anchoring pattern, the epi-fluorescence image and the photobleaching curve of 2xGFP molecules. (G–I) Construction and photobleaching analysis of 4xGFP molecules. (J) Diagram shows the deposition of 6xGFP on the surface of coverslips. Scale bar: 5 μM. (K) Wide-field epifluorescence images of 6xGFP on the surface of a coverslip. (L) Photobleaching curves of 6xGFP molecules, showing six photobleaching steps were clearly resolved. (M–Q) Construction and photobleaching analysis of 12xGFP molecules. To have a detailed view, the photobleaching steps of a 12xGFP molecule were separated into red and green boxed areas, as shown in O, P and Q. (R–V) Construction, imaging and the photobleaching steps of a 28xGFP molecule. (W) Intensity-based analyses of the copy number of GFPs in 6xGFP, 12xGFP and 28xGFP. (i) Distribution of intensities of single photobleaching steps collected from photobleaching curves of molecular complexes of GFPs (grey columns) fitted with a Gaussian function (red line). (ii–iv) Histograms (grey columns) fitted by Gaussian functions (red lines) show the distribution of initial intensities of photobleaching curves and the corresponding average copy numbers for 6xGFP, 12xGFP and 28xGFP constructs.

We first validated the ‘counting’ accuracy of SPEED microscopy using molecular complexes of GFP formed by controlled chemical coupling of GFP molecules. As shown in Fig. 1, molecular complexes containing one (1xGFP), two (2xGFP), four (4xGFP), six (6xGFP), twelve (12xGFP) and twenty-eight molecules of GFP (28xGFP) were constructed and used as standards. GFP-complexes were sparsely immobilized on the surface of PEG-coated coverslips, with a nearest neighboring distance of approximately 5 μm (Fig. 1 A–V). These well-separated fluorescent GFP-complexes enabled us to excite only one at a time in the illumination volume of SPEED microscopy (~320 nm in the x, y and z dimensions).

Second, we sought to collect complete photobleaching curves with all photobleaching steps for each GFP-complex used as standard. In principle, a *n*-step photobleaching curve was expected for each *n*xGFP construct assuming that all GFP molecules were initially fluorescent and could be photobleached individually and progressively over time by a laser beam. The number of GFP molecules per complex could then be determined from the number of steps in the photobleaching curve, but only if no *pre*-photobleaching occurred and if the GFP molecules were photobleached to become dark one-by-one.

**Figure 2:**
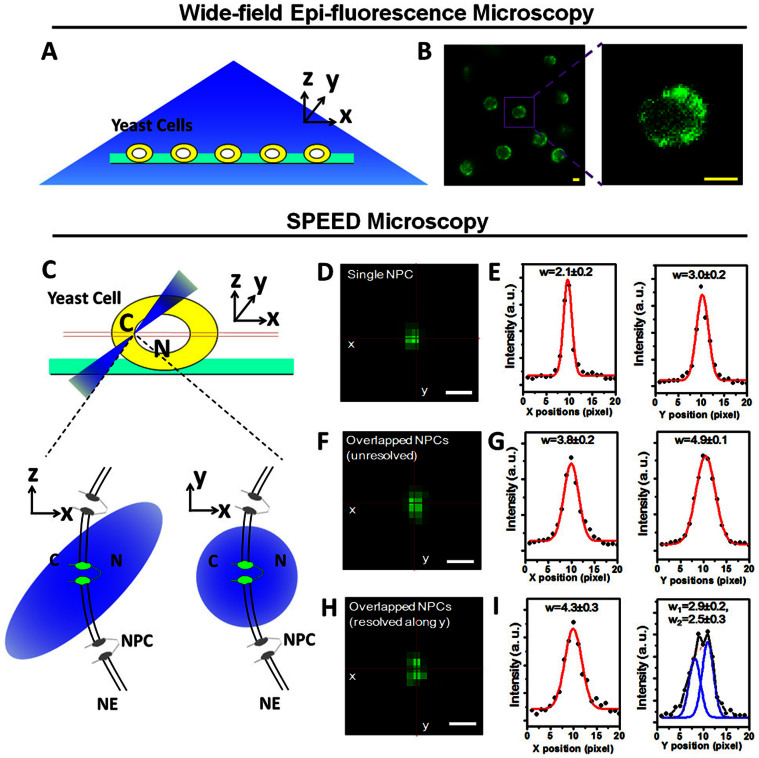
Single NPCs on the yeast NE illuminated by SPEED microscopy. (A) Diagram of yeast cells being imaged by wide-field epi-fluorescence microscopy. A Cartesian coordinate system is shown. (B) A typical epi-fluorescence image of yeast cells expressing a Nup-GFP fusion protein. The enlarged portion (right panel) shows the heterogeneous distribution of tagged NPCs on the NE of yeast. (C) SPEED microscopy illumination of a single NPC in a single yeast cell. Yeasts in growth medium were immobilized on concanavalin A-coated coverslips (green). C, cytoplasm; N, nucleus. Single GFP-labeled NPCs (green) on the NE were illuminated by an inclined diffraction-limit illumination point spread function (iPSF) of SPEED microscopy at the equatorial plane of the nucleus in the focal plane (between the double red lines). The iPSF forms an angle of 45° to the z direction. (D) A typical single GFP-tagged NPC observed by SPEED microscopy. (E) The fluorescent spot of the illuminated single GFP-NPC was fit well by an asymmetrical Gaussian function in *x* and *y* directions. The full width at half maximum (FWHM) of either fitting was shown in pixels. (F–G) Typical GFP-tagged overlapped NPCs observed by SPEED microscopy and unresolved by Gaussian fitting. (H–I) Typical GFP-tagged overlapped NPCs observed by SPEED microscopy and resolved by Gaussian functions in y direction.

Previous studies suggested that it would be challenging to detect multiple photobleaching steps from single fluorescent complexes of GFP molecules, and that low success rates for recording all photobleaching steps were expected^19^,^20^. The major challenges mentioned included the pre-photobleaching effect; the transient indistinguishable initial or intermediate photobleaching steps; the significant background noise; and the ability to distinguish photobleaching steps without artificial bias. To overcome these barriers, we optimized the detection of all photobleaching steps by SPEED microscopy as follows. First, to minimize the pre-photobleaching effect, we used a very weak excitation light to find individual molecular complexes of GFP on the surface of a coverslip, and then switched to a second laser beam with 1000-fold greater power to initiate the photobleaching steps (Methods). Second, to capture the fast-decaying initial or intermediate photobleaching steps, we used higher detection frame rates of 20–100 Hz, corresponding to 10–50 ms per frame. Third, to suppress the background noise, our SPEED microscope minimized the excitation volume by using the inclined, diffraction-limited illumination volume formed by the focal plane after guiding the laser beam through the edge of the objective (Fig. 2). This design dramatically reduced the out-of-focus fluorescence and also completely avoided the auto-fluorescence in the center of the objective. Compared to conventional wide-field microscopy, SPEED microscopy achieves a much higher (≥11) signal-to-noise (S/N) ratio for single-particle localization and provides a capacity of capturing single NPCs on the NE of live yeast (Fig. 2)^17^,^18^,^21^. Additionally, the background fluorescence is subtracted from the fluorescence signal of GFPs in our measurements to further suppress the effects of background noise. Lastly, to identify each photobleaching step without arbitrary selection, we employed a published computer algorithm that resolves single steps in photobleaching curves based on the maximum likelihood ratio method^22^. The details were described in Supplementary Materials.

As shown in Fig. 1, these technical advances enabled us to clearly identify 1, 2, 4, 6, 12 and 28 photobleaching steps in the photobleaching curves of the 1xGFP, 2xGFP, 4xGFP, 6xGFP, 12xGFP and 28xGFP molecular standards, respectively (Fig. 1). We even identified four initial transient decaying steps in the photobleaching curves of 28xGFP (Fig. 1U), which were later confirmed by the independent intensity-based measurements discussed below.

To complement the direct-counting approach, we also calculated the total initial number of GFP molecules in each complex, by dividing the initial fluorescence intensity of the complex (at time 0 of its photobleaching curve) by the averaged intensity of an individual phototbleaching step (Fig. 1W). For example, over 700 well-resolved photobleaching steps were used to calculate the averaged intensity of one GFP photobleaching step (Fig. 1W–i). Likewise, the distribution of initial fluorescence intensities for the 6xGFP, 12xGFP and 28xGFP complexes were plotted to calculate the copy numbers of GFPs in each GFP-complex. For 6xGFP and 12xGFP, the calculated copy numbers were consistent with the maximum copy numbers determined from the photobleaching curves. For the 28xGFP complexes, the calculated copy number from the fluorescence intensity was twenty eight, in agreement with the designed copy number of the 28xGFP complex as well. This pointed to the existence of four brief initial photobelaching steps in the direct counting approach used for the 28xGFP complex (Fig. 1U).

We concluded from the above experiments that SPEED microscopy can be used to directly count up to twenty-eight GFP molecules in a complex based on its photobleaching curve. We also concluded that the copy number of GFPs in a complex can be calculated independently, from the ratio of the initial fluorescence intensity of GFP-complexes over the emission intensity of one GFP molecule. Next, we adapted both approaches to directly determine the copy number of Nups within individual NPCs of live yeast.

### SPEED microscopy illumination of individual NPCs on the NE of live yeast cells

**Figure 3:**
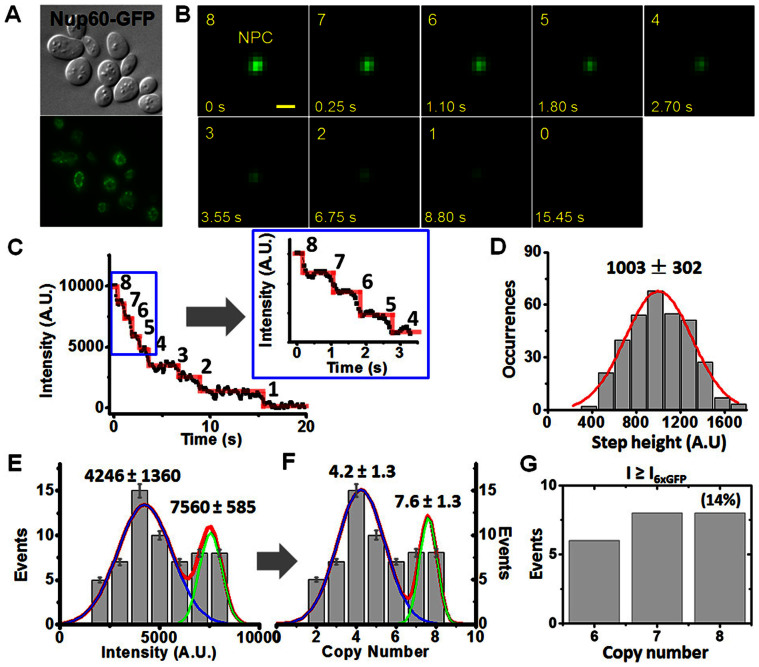
Counting the copy-number of Nup60-GFP in yeast NPCs. (A) Yeasts expressing GFP-tagged Nup60 were observed in either bright-field or fluorescence mode of epi-fluorescence microscopy. (B) The sequential photobleaching images of Nup60-GFPs located in a single NPC over time by SPEED microscopy. Scale bar: 1 μm. (C) A typical photobleaching curve for a Nup60-GFP labeled NPC in yeast. Eight GFP-Nup60 photobleaching steps were resolved corresponding to eight molecules of Nup60 per NPC. The first several transient steps were enlarged for a detailed look (inset). (D) Distribution of intensities of single photobleaching steps collected from photobleaching curves of Nup60-GFPs (grey columns) fitted with a Gaussian function (red line). (E–F) Distributions of the initial intensities of photobleaching curves and the corresponding copy-number of Nup60-GFP per NPC. Both histograms were best-fitted by two Gaussian functions that generated two clustered intensities and two average copy-numbers (blue, green and red lines). (G) The copy numbers of Nup60-GFP per NPC directly counted from the photobleaching steps of Nup60-GFP labeled NPCs that have the initial intensities equal to and bigger than six-fold of the intensity of single Nup60-GFP in live yeast cells. Approximately 14% of all measured NPCs enabled us to obtain a maximum copy number of eight for Nup60-GFP per NPC.

Yeast cells expressing GFP-tagged Nups were first imaged using a wide-field epi-fluorescence microscope (Fig. 2 A–B). The cells were alive during the microscopic analyses (Supplemental Fig. S1). Sparse and overlapped fluorescent spots corresponding to GFP-labeled NPCs were observed (Fig. 2B and Fig. 3A). NE areas devoid of NPCs were also observed, as expected^23^.

Previously SPEED microscopy was used to image a single fluorescent NPC on the NE of permeabilized or live HeLa cells^17^,^18^,^21^. The success was based on the fact that SPEED microscopy provided an inclined illumination point spread function (iPSF) with ~320 nm in the x, y and z dimensions, which is smaller than the averaged nearest neighboring distance between NPCs in HeLa cells, after an incident 488-nm laser beam used to illuminate Nup-GFP was shifted ~237 μm off the center of the objective (Fig. 2C)^17^,^18^. However, imaging of a single fluorescent NPC in live yeast could be more challenging since yeast has a smaller nucleus and a higher spatial density of NPCs on the NE than HeLa cells. Fortunately, the NPCs on yeast NE are not evenly distributed and their spatial density ranges from 4.0 to 22.0 NPCs/μm^2^ 23. Thus, to avoid imaging the NE areas with visibly-high NPC density, we specifically selected the regions on the NE with sparse fluorescent spots of NPCs. As a result, only isolated GFP-NPCs that were visible on the equator of the NE were brought into the small inclined diffraction-limit illumination volume of SPEED microscopy (Fig. 2 C–D).

As done previously^17^,^18^,^21^, 2D Gaussian function fittings were employed to test whether the fluorescent spot of NPC captured on the NE is an overlap of multiple NPCs, or a single NPC (Fig. 2 D–E). Specifically, the full width at half maximum (FWHM) of the selected fluorescent NPC spots in the long and short axes of the fluorescence spot was analyzed by 2D asymmetrical Gaussian function fitting. The width in *x* and *y* directions of a single GFP-NPC on the NE of yeast should be smaller or equal to 580 nm and 790 nm (≤2.4 and ≤3.3 pixels as shown in Fig. 2 D–E and supplemental Fig. S2) assuming there are 8, 16 or 32 copies of Nups in each NPC (supplemental Fig. S2)^17^. Therefore, any fluorescent spots on the yeast NE with unresolved larger FWHM widths (Fig. 2 F–G) or with resolved multiple peaks (Fig. 2 H–I) were not analyzed further. Only single isolated NPCs were selected for further analyses (supplemental Fig. S2). Finally, for the selected single fluorescent NPCs, the excitation laser beam in the SPEED microscope was kept on until all of the GFP fluorescence from the illuminated single GFP-NPC was completely photobleached (Fig. 3).

### Counting the copy-number of Nups in individual yeast NPCs

We adapted the approach described above to count the copy number of each Nup *in situ* using live yeast NPCs. For the analysis, we used haploid *S. cerevisiae* strains featuring NUP-GFP gene fusions constructed by homologous recombination at chromosomal loci. These were ideal because they are expressed at endogenous levels from endogenous NUP promoters^24^,^25^, and because all cellular copies of the corresponding Nup contained a GFP label. In the few cases where the GFP label interfered with Nup localization or with cell viability, the Nup was excluded from the analysis. In the end, 24 of ~30 Nups were analyzed (Supplemental Fig. S3).

**Figure 4:**
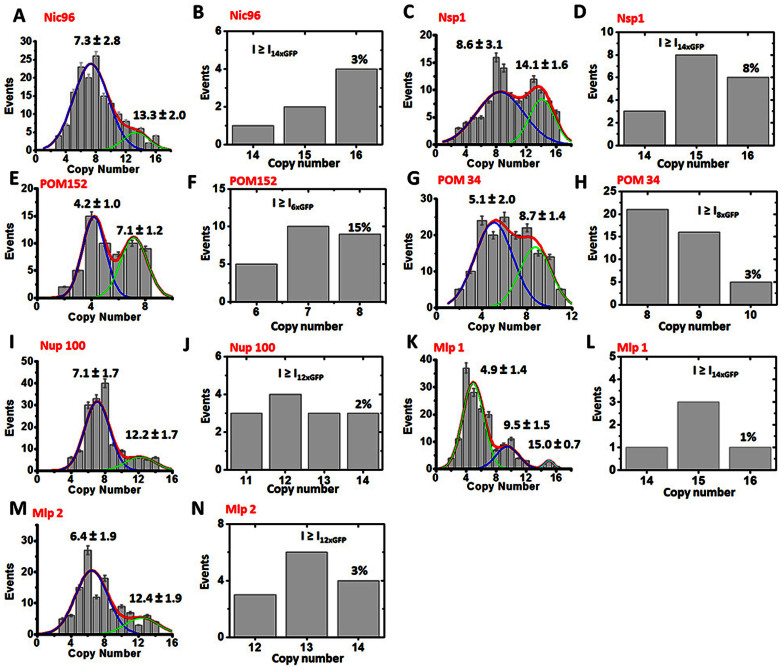
Copy numbers of Nups in the yeast NPCs obtained from either the initial intensities or from the direct counted steps in photobleaching curves. (A) Histogram of copy numbers of Nic96-GFP per NPC achieved from the initial intensities of Nic96-GFP molecules per NPC divided by the intensity of a single Nic96-GFP molecule. Gaussian fittings (red line) of the histogram yielded two averaged copies of 7.3 ± 2.8 (blue) and 13.3 ± 2.0 (green). (B) Direct counted copy numbers of Nic96-GFP per NPC. Approximately 3% of all the scanned fluorescent NPCs that have initial intensities equal to or bigger than that of fourteen copies of Nic96-GFP molecules generated a maximum copy number of 16. (C–D) The average intensity-based copy numbers and the photobleaching-step-based maximum copy of Nsp1-GFP per yeast NPC. (E–F) The intensity-based averaged copy numbers and the photobleaching-step-based maximum copy of POM152-GFP in yeast NPC. (G–H) The averaged intensity-based copy numbers and the photobleaching-step-based maximum copy of POM34-GFP per NPC. (I–J) The intensity-based averaged copy numbers and the photobleaching-step-based maximum copy of Nup100-GFP per NPC. (K–L) The intensity-based averaged copy numbers and the photobleaching-step-based maximum copy of Mlp1-GFP per NPC. (M–N) The intensity-based averaged copy numbers and the photobleaching-step-based maximum copy of Mlp2-GFP per NPC.

For each live yeast strain analyzed, we collected 60–160 photobleaching curves from 60–160 individual fluorescent NPCs (Figs. 3 and 4, Table 1). Remarkably, in contrast to the one average copy number observed for each synthetic GFP oligomer standard (Fig. 1), two or three average copy numbers were observed for each GFP-Nup in live yeast NPCs. For example, in the case of NPCs labeled with Nup60-GFP (Fig. 3A), the initial intensities of the photobleaching curves collected from the 60 NPCs generated two clustered distributions. After dividing each by the averaged intensity of single photobleaching steps for Nup60-GFP (Fig. 3 C–D), two different copy numbers, 4.2 ± 1.3 and 7.6 ± 1.3, were calculated (Fig. 3 B–C). Consistently, we detected eight photobleaching steps in 14% of all the sampled NPCs (Fig. 3 C and G), suggesting eight Nup60-GFP molecules maximum per NPC. Thus, the fluorescence intensity-based calculation method and the direct photo-bleaching step counting method showed a maximum copy of eight Nup60-GFPs in live yeast NPCs. Similarly, in the case of Nup49-GFP labeled NPCs, the initial intensities of NPC fluorescence in the photobleaching curves suggested two different copy numbers, 9.7 ± 4.9 and 15.8 ± 0.8 (data not shown). In addition, approximately 9% of all the measured NPCs revealed a maximum of sixteen copies of Nup49-GFP per NPC based on the photobleaching step counting method (Table 1).

The observed maximum copy-number of 8 and 16 for for Nup60 and Nup49 respectively, was consistent with previous estimates of their stoichiometry^1^,^11^. In contrast, the lower copy-numbers detected of approximately 4 molecules of Nup60-GFP and 10 for Nup49-GFP were initially surprising, but in retrospect, should have been expected for a number of reasons. First, not all Nup-GFP molecules synthesized in live yeast fluoresce. Previous studies have shown that up to 20% of GFP molecules in chimeras do not fold well-enough to emit fluorescence due to interference of adjacent domains with folding^26^,^27^, and/or may experience delays in chromophore centre maturation^28^. Another possibility is that not every NPC in live yeast contains a maximum copy number of each Nup. For example, some Nups have residence times in the NPC that last only seconds to minutes, potentially leaving several binding sites unoccupied at any given moment^14^. To determine if any of these possibilities affect the accuracy of our measurements, we conducted further tests.

To minimize the effect of non-fluorescent GFP molecules, we scanned thousands of fluorescent NPCs in hundreds of live yeast and included 60–160 well-isolated NPCs in 60–160 different cells for each analysis. These large-numbers increased the chances of detecting the maximum copy number for each Nup in NPCs. Next, we tested whether the maximum number of Nups visible per NPC can be affected by the rapid Nup dissociation or association events during sampling. Fortunately, we found that the average NPC dissociation or association time for yeast Nups is>10 s (unpublished data), which is orders of magnitude longer than the detection time of 10–50 ms for each point in the photobleaching curve. This makes it unlikely that a GFP-Nup could have dissociated or associated during sampling time of the first data point (time 0). Also, copy-numbers for each Nup were derived from many single NPC data, so the net effect of opposite association and dissociation processes should have canceled each other during our detections. For these reasons we believe that the copies of GFP-Nups detected here per NPC by SPEED microscopy were not under or overestimated.

Following the procedure above, the maximum copy numbers of twenty four different Nups within the NPC of live yeast were determined (Fig. 4 and Table 1). Each NPC contained: a maximum of sixteen copies of Nic96 (Fig. 4 A–B) and Nsp1 (Fig. 4 C–D), rather than thirty two copies as previously estimated^1^,^11^; approximately sixteen copies of Gle2, Mlp1 (Fig. 4 K–L), Nup49, Nup53, Nup57, Nup82, Nup84, Nup116, Nup133, Nup145C, Nup170 and Nup188; eight copies of Nup1, Nup60, Nup159 and Pom152 (Fig. 4 E–F); and ten to fifteen copies of Nup59, Pom34 (Fig. 4 G–H), Nup157, Nup192, Nup100 (Fig. 4 I–J), and Mlp2 (Fig. 4 M–N). The copy-number of other Nups (Gle1, Ndc1, Nup2, Nup42, Nup85, Nup120, Nup145N and Pom33)^29^ was not listed because suitable haploid strains expressing their corresponding GFP fusions were not available at the time of this analysis.

## Discussion

The maximum copy-number of Nups per NPC observed here in live yeast by SPEED microscopy provides new insight into the subunit stoichiometry of NPCs. The copy-number of two Nups, which were never before estimated (Mlp1 and Mlp2) was recorded here at fourteen to sixteen copies per NPC (Fig. 4 K–N). For fourteen of twenty four other Nups analyzed, the maximum copy-number per NPC measured here was in agreement with (and thus validated) previous estimates of 16 copies of Gle2, Nup49, Nup53, Nup57, Nup82, Nup84, Nup116, Nup133, Nup145C, Nup170, Nup188; and 8 copies of Nup1, Nup60 and Nup159^1^,^11^. Notably, contrary to previous estimates, we found that none of the yeast Nups are present in 32 copies per NPC as was predicted for Nsp1, Nic96 and Pom34^1^,^11^. Lastly, for Nic96, Nsp1 and Pom152 the number of molecules counted was only half of what was previously estimated.

Interesting deviations were observed from the expected eight-fold symmetry pattern of NPCs. For example, Pom34 was detected at ~10 copies per NPC; and Nup59, Nup100, Nup157, Nup192 and Mlp2 were detected at ~13 to 15 copies per NPC. In all likelihood, this is due to a competition of duplicate Nups for shared or overlapping docking sites at the NPC. Indeed, five of these ‘stoichiometric misfits’ (i.e. Nup59, Nup100, Nup157, Nup192 and Mlp2) are in fact gene duplicates of Nup53, Nup116, Nup170, Nup188, and Mlp1, respectively^30^,^31^,^32^,^33^, and could very likely compete with their homolog for the same docking site at the NPC, a possibility that can be tested in the future. Alternatively, there are reported cases where nine-fold or ten-fold symmetry of NPC was noted^34^,^35^.

The current estimate of the copy number of Nups in human NPCs may also need to be revised. It was recently suggested that Nup copy numbers vary considerably between different human cell lines, and that approximately one third of human Nups are present at 32 copies per NPC^16^, much higher than the average copy number of 16 for yeast NPCs. This, and the fact that human Nups have longer amino acid sequences, explains why the yeast and human NPCs are so different in size, despite being composed of ~32 different Nups each yeast NPC^1^,^4^,^5^ Our future interest is to adapt this SPEED microscopy approach to count the copy number of Nups in NPCs of different human cell lines.

In conclusion, the technical advances of SPEED microscopy described here enabled us to directly count the number of subunits of a supramolecular structure in living cells. This real-time and highly-accurate determination of subunit stoichiometry forces a refinement of current 3D-architecture maps of the yeast NPC. These refined maps advance our understanding of the structure, function, composition-dynamics, and biogenesis of NPCs. More generally, our molecular counting technology can be adapted to investigate the subunit composition of other supramolecular structures in yeast and higher eukaryotes.

## Methods

### Yeast strains and growth conditions

The *Saccharomyces cerevisiae* strains used were generated by Huh *et al.*^24^ with the exception of the Nup116-GFP expressing strain, which was obtained as a gift from *S. Simon* lab^36^. In all cases (except for Nup116-GFP) the GFP-tag was added at the C-terminus of the Nup. Yeast were grown in liquid YPD medium (1% yeast extract, 2% peptone, 2% dextrose) at 30°C to a cell density of 0.5–2.0 OD_600_/mL. For live-cell imaging, the cells were harvested by centrifugation, resuspended in minimal medium (0.17% yeast nitrogen base, 0.5% ammonium sulfate, 0.079% CSM), and immobilized on concanavalin A-coated coverslips. Yeast cells continued to grow in this media during the observation time (~90 min; Supplemental Fig. S1). The duration of each cell measurement was less than 2 min. Yeast were kept in fresh media for less than 60 min when measuring fluorescence overall. All measurements were conducted on visibly-live cells.

### Microscopy

Live yeast expressing GFP-tagged nucleoporins were first observed using a wide-field fluorescence microscope equipped with a charge-coupled device camera (Coolsnap HQ2, Photometrics) to locate the nucleus equator. Then the selected area was observed under the SPEED microscope, which includes an Olympus IX81 instrument equipped with a 1.4 NA 100× oil-immersion apochromatic objective (UPLSAPO 100X, Olympus), a 120 mW ArKr tunable ion laser (Melles Griot), an on-chip multiplication gain charge-coupled device camera (Cascade 128+, Roper Scientific), and the Slidebook software package (Intelligent Imaging Innovations) for data acquisition and processing. Excitation was provided by a 488 nm laser. Green fluorescence emissions were collected through the same objective, filtered by a dichroic filter (Di01-R405/488/561/635-25x36, Semrock) and an emission filter (NF01-405/488/561/635-25X5.0, Semrock), and imaged by the CCD camera. Images were captured continuously; the exposure time of each image was 10–50 ms.

### Construction of nxGFP molecular complexes

**1xGFP:** GFP was genetically enginered with N-terminal 6xHis-tags and then overexpressed in *Escherichia coli* BL21(λDE3) strain by 1 mM IPTG induction. Branson Sonic Dismembrator 550 was used 2 times at 5 minutes with 50% duty cycle and output 3 to break up the cells. The extract was further purified by Superflow (Qiagen, Valencia, CA), MonoQ, and Superdex 200 (Amersham Pharmacia) chromatography. The three chromatographic steps were necessary to yield a single band by Coomassie-stained polyacrylamide gel electrophoresis. **2xGFP:** Genetically engineered 2xGFP^37^ was expressed from a pTrcHisB vector in JM109 *E. Coli* at 30°C and was purified to homogeneity from cell lysates using nickel-nitrilotriacetic Superflow (Qiagen, Valencia, CA), SAX-10, and SEC-1 (Thermo Scientific) chromatography. The purified 1xGFP and 2xGFP molecules were diluted to a picomolar concentration, deposited on slides (the natural charge allowed these GFPs to adhere to the surface of the glass), and examined by SPEED microscopy (Fig. 1 A–F). **4xGFP:** Purified 2xGFP was crosslinked to itself by incubating two equal molar concentrations of 2xGFP with succinimidyl-6-hydrazino-nicotinamide (S-HyNic) and succinimidyl-4-formylbenzamide (S-4FB), respectively (Solulink, San Diego, CA). The S-4FB NS S-HyNic linkers conjugate to proteins through primary amines (-NH_2_) on lysines or the N-terminus. HyNic-modified 2xGFP was incubated with 4FB-modified 2xGFP in a catalyzed conjugation reaction, which resulted in two 2xGFP molecules conjugating through a UV-traceable, exceptionally-strong bond (bisoarylhydrazone) with measurable absorbance at 354 nm (Fig. 1 G–I). 2xGFP dimers (4xGFP) were resolved from 2xGFP and GFP multimers by ion exchange chromatography. Purity was confirmed by SDS-PAGE analysis. **6xGFP:** To create 6xGFP complexes, we used the homotetrameric streptavidin molecule (Sigma S4762-5MG) to bridge one biotin molecule on a slide surface to three biotinylated 2xGFP molecules in solution. First, we coated the surface of 2xGFP with a modified biotin molecule featuring a long-chain PEG3 linker (Solulink, San Diego, CA); this linker was included to reduce crowding of 2xGFP molecules on the streptavidin surface. We then generated slides with an average biotin surface density of 10^9^ molecules/cm^2^ (Microsurfaces Inc, NJ); this distribution in turn limited the average nearest-neighbor distance of 6xGFP complexes to ~5 μm. This distance could be increased further by including streptavidin molecules that had been pre-incubated with a 1:3 molar equivalent of biotin. These streptavidin-biotin complexes (10 μg/ml in PBS buffer) were added to biotin coated slides, followed by unreacted streptavidin molecules and incubation for 30 min in a humidified chamber. After washing the slide (with PBS, 50 mM Tween-20) to remove unbound streptavidin, biotinylated 2xGFP molecules (in PBS buffer, 10% glycerol) were added at nanomolar concentration and the slide was incubated 30 min further in a humidified chamber at room temperature. Unbound, biotinylated 2xGFP molecules were removed by washing as before. These slides were then examined by SPEED microscopy (Fig. 1 J–L). **12xGFP:** To create 12xGFP complexes, we employed the same procedure as described for 6xGFP, but we used 4xGFP as substrate instead of 2xGFP (Fig. 1 M–Q). **28xGFP:** To create 28xGFP complexes we followed the same procedure as for 6xGFP and 12xGFP, but used 2xstreptavidin molecules instead. The 2xstreptavidin conjugates w generated using the same crosslinking procedure described for 4xGFP. 2xstreptavidin complexes bound to biotin-coated slides had up to 7 available sites to bind biotinylated 4xGFP, thus capturing a maximum of 28 copies of GFP per complex (Fig. 1 R–V). Only the brightest, single fluorescent spots were examined (Fig. 1S); dimmer spots contained fewer than the maximum number of 4xGFP substrates bound.

### Identification of single NPCs on the yeast nuclear envelope

Two steps were followed to select single NPCs oriented perpendicularly to the NE on the equator of the nucleus, and to the *y* direction of the Cartesian coordinates (*x*, *y*) in the CCD camera. First, a fluorescent NPC ‘spot’ on the nuclear equator was chosen such that the tangent of the NE at the location of this NPC spot was parallel to the *y* direction of the Cartesian coordinates (*x*, *y*) in the CCD camera. Second, the FWHM in the long and short axes of the fluorescence spot was analyzed by 2D asymmetrical Gaussian function fitting, as shown in supplemental Fig. S2. As a result, any spots with wider FWHM (Fig. 2 F–G) or with resolved peaks (Fig. 2 H–I) were not analyzed further.

### Minimization of the pre-photobleaching effect

Control experiments showed that pre-photobleaching of the GFP-Nup fluorescence during the lamp illumination time in the wide-field microscope (prior to SPEED microscopy) was unlikely. A lamp power density of 0.18 W/cm^2^ was used in the wide-field epifluorescence microscope to image the fluorescent NEs of live yeast and the search time needed to locate individual GFP-labeled NPCs on the NE was only 10–20 s. This was much shorter than the recorded photobleaching times of approximately 350 s for the first GFP out of 8 GFP-Nups, or about 70 s for the first GFP in the case of 16 copies of a GFP-Nup. Thus, pre-photobleaching of GFP fluorescence was maximally avoided during the search process. In contrast, the laser power density of 250 W/cm^2^ used subsequently for the SPEED microscopy analysis made the average photobleaching time of the first GFP-Nup very short (200 ± 63 ms) for cases where 8 copies of a GFP-Nup were detected, and even shorter (100 ± 31 ms) when 16 copies of a GFP-Nup were detected.

## Supplementary Material

Supplementary Information Supplementary Info

## Figures and Tables

**Table 1:** Stoichiometry of Nups in the yeast NPC

*S. cerevisiae* Nups	*In vitro* estimated copies per NPC	*In vivo* visualized, maximum copies per NPC*
Gle2	16^1^,^11^	16 (2%, 120)
Mlp1	ND	16 (1%, 160)
Mlp2	ND	14 (3%, 120)
Nic96	32^1^,^11^	16 (3%, 160)
Nsp1	32^1^,^11^	16 (8%, 120)
Nup1	8^1^,^11^	8 (9%, 120)
Nup49	16^1^,^11^	16 (9%, 120)
Nup53	16^1^,^11^	16 (6%, 120)
Nup57	16^1^,^11^	16 (4%, 120)
Nup59	16^1^,^11^	14 (4%, 120)
Nup60	8^1^,^11^	8 (14%, 60)
Nup82	16^1^,^11^	16 (7%, 120)
Nup84	16^1^,^11^	16 (7%, 120)
Nup100	8^1^, 16^11^	14 (2%, 160)
Nup116	16^1^	16 (2%, 120)
Nup133	16^1^,^11^	16 (9%, 120)
Nup145C	16^1^,^11^	16 (10%, 120)
Nup157	16^1^,^11^	14 (4%, 120)
Nup159	8^1^,^11^	8 (30%, 60)
Nup170	16^1^,^11^	16 (2%, 120)
Nup188	16^1^,^11^	16 (10%, 120)
Nup192	16^1^,^11^	15 (2%, 120)
Pom34	16^11^, 32^1^	10 (3%, 160)
Pom152	16^1^,^11^	8 (15%, 60)

*Shown in brackets is the percentage of photobleaching curves that produced the maximum copy-number of Nups per NPC listed (i.e. the incidence of occurrence), followed by the number of NPCs sampled in each case. Highlighted in gray are significant differences between previous estimates and our current determinations. ND, not detected.

## References

[b1] Rout M. P. *et al.* The yeast nuclear pore complex: composition, architecture, and transport mechanism. J. Cell Biol. 148, 635–651 (2000). 10684247 10.1083/jcb.148.4.635 PMC2169373

[b2] Cronshaw J. M., Krutchinsky A. N., Zhang W., Chait B. T. & Matunis M. J. Proteomic analysis of the mammalian nuclear pore complex. J. Cell Biol. 158, 915–927 (2002). 12196509 10.1083/jcb.200206106 PMC2173148

[b3] Akey C. W. & Radermacher M. Architecture of the Xenopus nuclear pore complex revealed by three-dimensional cryo-electron microscopy. J. Cell Biol. 122, 1–19 (1993). 8314837 10.1083/jcb.122.1.1 PMC2119598

[b4] Yang Q., Rout M. P. & Akey C. W. Three-dimensional architecture of the Isolated yeast nuclear pore complex: functional and evolutionary implications. Mol. Cell 1, 223–234 (1998). 9659919 10.1016/s1097-2765(00)80023-4

[b5] Kiseleva E. *et al.* Yeast nuclear pore complexes have a cytoplasmic ring and internal filaments. J. Struct. Biol. 145, 272–288 (2004). 14960378 10.1016/j.jsb.2003.11.010

[b6] Maimon T., Elad N., Dahan I. & Medalia O. The human nuclear pore complex as revealed by cryo-electron tomography. Structure 20, 998–1006 (2012). 22632834 10.1016/j.str.2012.03.025

[b7] Beck M. *et al.* Nuclear pore complex structure and dynamics revealed by cryoelectron tomography. Science 306, 1387–1390 (2004). 15514115 10.1126/science.1104808

[b8] Beck M., Lucic V., Foerster F., Baumeister W. & Medalia O. Snapshots of nuclear pore complexes in action captured by cryo-electron tomography. Nature 449, 611–615 (2007). 17851530 10.1038/nature06170

[b9] Bui K. H. *et al.* Integrated Structural Analysis of the Human Nuclear Pore Complex Scaffold. Cell 155, 1233–1243 (2013). 24315095 10.1016/j.cell.2013.10.055

[b10] Alber F., Förster F., Korkin D., Topf M. & Sali A. Integrating Diverse Data for Structure Determination of Macromolecular Assemblies. Annu. Rev. Biochem. 77, 443–477 (2008). 18318657 10.1146/annurev.biochem.77.060407.135530

[b11] Alber F. *et al.* Determining the architectures of macromolecular assemblies. Nature 450, 683–694 (2007). 18046405 10.1038/nature06404

[b12] Alber F. *et al.* The molecular architecture of the nuclear pore complex. Nature 450, 695–701 (2007). 18046406 10.1038/nature06405

[b13] Dilworth D. J. *et al.* Nup2p Dynamically Associates with the Distal Regions of the Yeast Nuclear Pore Complex. J. Cell Biol. 153, 1465–1478 (2001). 11425876 10.1083/jcb.153.7.1465 PMC2150724

[b14] Rabut G., Doye V. & Ellenberg J. Mapping the dynamic organization of the nuclear pore complex inside single living cells. Nat. Cell Biol. 6, 1114–1121 (2004). 15502822 10.1038/ncb1184

[b15] Löschberger A. *et al.* Super-resolution imaging visualizes the eightfold symmetry of gp210 proteins around the nuclear pore complex and resolves the central channel with nanometer resolution. J. Cell Sci. 125, 570–575 (2012). 22389396 10.1242/jcs.098822

[b16] Ori A. *et al.* Cell type-specific nuclear pores: a case in point for context-dependent stoichiometry of molecular machines. Mol Syst. Biol. 9, 648 (2013). 23511206 10.1038/msb.2013.4 PMC3619942

[b17] Ma J. & Yang W. Three-dimensional distribution of transient interactions in the nuclear pore complex obtained from single-molecule snapshots. Proc. Natl. Acad. Sci. USA 107, 7305–7310 (2010). 20368455 10.1073/pnas.0908269107 PMC2867735

[b18] Ma J., Goryaynov A., Sarma A. & Yang W. Self-regulated viscous channel in the nuclear pore complex. Proc. Natl. Acad. Sci. USA 109, 7326–7331 (2012). 22529346 10.1073/pnas.1201724109 PMC3358865

[b19] Jiang Y. *et al.* Sensing cooperativity in ATP hydrolysis for single multisubunit enzymes in solution. Proc. Natl. Acad. Sci. USA 108, 16962–16967 (2011). 21896715 10.1073/pnas.1112244108 PMC3193194

[b20] Gordon M. P., Ha T. & Selvin P. R. Single Molecule High Resolution Imaging with Photobleaching. Proc. Nat. Acad. Sci. USA. 101, 6462–6465 (2004). 15096603 10.1073/pnas.0401638101 PMC404067

[b21] Ma J. *et al.* High-resolution three-dimensional mapping of mRNA export through the nuclear pore. Nat. Comm. 4, 2414 (2013). 10.1038/ncomms3414 PMC3800679 24008311

[b22] Watkins L. P. & Yang H. Detection of Intensity Change Points in Time-Resolved Single-Molecule Measurements. J. Phys. Chem. B. 109, 617–628 (2005). 16851054 10.1021/jp0467548

[b23] Winey M., Yarar D., Giddings T. H. J. & Mastronarde D. N. Nuclear Pore Complex Number and Distribution throughout the Saccharomyces cerevisiae Cell Cycle by Three-Dimensional Reconstruction from Electron Micrographs of Nuclear Envelopes. Mol. Biol. Cell 8, 2119–2132 (1997). 9362057 10.1091/mbc.8.11.2119 PMC25696

[b24] Huh W. K. *et al.* Global analysis of protein localization in budding yeast. Nature 425, 686–691 (2003). 14562095 10.1038/nature02026

[b25] Ghaemmaghami S. *et al.* Global analysis of protein expression in yeast. Nature 425, 737–741 (2003). 14562106 10.1038/nature02046

[b26] Tanudji M., Hevi S. & Chuck S. L. Improperly folded green fluorescent protein is secreted via a non-classical pathway. J. Cell Sci. 115, 3849–3857 (2002). 12235295 10.1242/jcs.00047

[b27] Chang H. C., Kaiser C. M., Hartl F. U. & Barral J. M. De novo folding of GFP fusion proteins: high efficiency in eukaryotes but not in bacteria. J. Mol. Biol. 353, 397–409 (2005). 16171814 10.1016/j.jmb.2005.08.052

[b28] Pouwels L. J., Zhang L., Chan N. H., Dorrestein P. C. & Wachter R. M. Kinetic isotope effect studies on the de novo rate of chromophore formation in fast- and slow-maturing GFP variants. Biochemistry 47, 10111–10122 (2008). 18759496 10.1021/bi8007164 PMC2643082

[b29] Chadrin A. *et al.* Pom33, a novel transmembrane nucleoporin required for proper nuclear pore complex distribution. J Cell Biol. 189, 795–811 (2010). 20498018 10.1083/jcb.200910043 PMC2878943

[b30] Fabre E. & Hurt E. Yeast genetics to dissect the nuclear pore complex and nucleocytoplasmic trafficking. Annu. Rev. Genet. 31, 277–313 (1997). 9442897 10.1146/annurev.genet.31.1.277

[b31] Hawryluk-Gara L. A., Shibuya E. K. & Wozniak R. W. Vertebrate Nup53 Interacts with the Nuclear Lamina and Is Required for the Assembly of a Nup93-containing Complex. Mol. Biol. Cell 16, 2382–2394 (2005). 15703211 10.1091/mbc.E04-10-0857 PMC1087243

[b32] Devos D. *et al.* Simple fold composition and modular architecture of the nuclear pore complex. Proc. Natl. Acad. Sci. USA 103, 2172–2177 (2006). 16461911 10.1073/pnas.0506345103 PMC1413685

[b33] Aitchison J. D. & Rout M. P. The yeast nuclear pore complex and transport through it. Genetics 190, 855–883 (2012). 22419078 10.1534/genetics.111.127803 PMC3296253

[b34] Franke W. W. Isolated nuclear membranes. J. Cell Biol. 31, 619–623 (1966). 5339525 10.1083/jcb.31.3.619 PMC2107077

[b35] Hinshaw J. E. & Milligan R. A. Nuclear pore complexes exceeding eightfold rotational symmetry. J. Struct. Biol. 141, 259–268 (2003). 12648571 10.1016/s1047-8477(02)00626-3

[b36] Mattheyses A., Kampmann M., Atkinson C. & Simon S. Fluorescence anisotropy reveals order and disorder of protein domains in the nuclear pore complex. Biophys. J. 99, 1706–1717 (2010). 20858414 10.1016/j.bpj.2010.06.075 PMC2941012

[b37] Yang W., Gelles J. & Musser M. S. Imaging of single-molecule translocation through nuclear pore complexes. Proc. Natl. Acad. Sci. USA 101, 12887–12892 (2004). 15306682 10.1073/pnas.0403675101 PMC516490

