# Supplementary material for: Quantifying Nucleoporin Stoichiometry Inside Single Nuclear Pore Complexes In vivo
Source: Sci Rep. 2015 Mar 23;5:9372. doi: 10.1038/srep09372 (PMC4894440; doi:10.1038/srep09372)
Supplement: Supplementary Information — Supplementary Info [file srep09372-s1.pdf]

Supplementary Information

Quantifying Nucleoporin Stoichiometry Inside Single Nuclear Pore Complexes In vivo

Lan Mi[1,3], Alexander Goryaynov[1], Andre Lindquist[2], Michael Rexach[2,4] & Weidong Yang[1,4]

[1]Department of Biology, Temple University, Philadelphia, PA 19122

[2]Department of Molecular, Cell and Development Biology, University of California, Santa Cruz, CA 95064

[3]Department of Optical Science and Engineering, Shanghai Engineering Research Center of Ultra-Precision Optical Manufacturing, Fudan University, Shanghai 200433, China

[4]Correspondence should be addressed to: Department of Biology, Temple University, Philadelphia, PA 19122. Tel.: +1-215-204-2312; Fax: +1-215-204-6646 ; Email: weidong.yang@temple.edu Or Department of Molecular, Cell and Development Biology, University of California, Santa Cruz, CA 95064. Tel.: +1-831-459-4986 ; Fax: +1-831-459-3139 ; Email: rexach@biology.ucsc.edu.

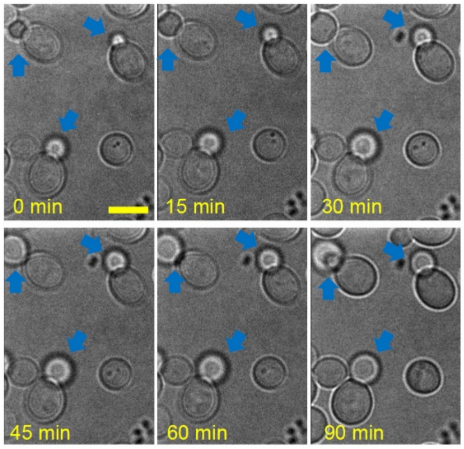

Supplemental Figure S1 | Yeast continued to grow during microscopic analysis. Yeast cells in growth medium were immobilized on concanavalin A-coated coverslips. New cell buds (blue arrows) grew in size over time relative to the mother cell. Scale bar, 5 μm.

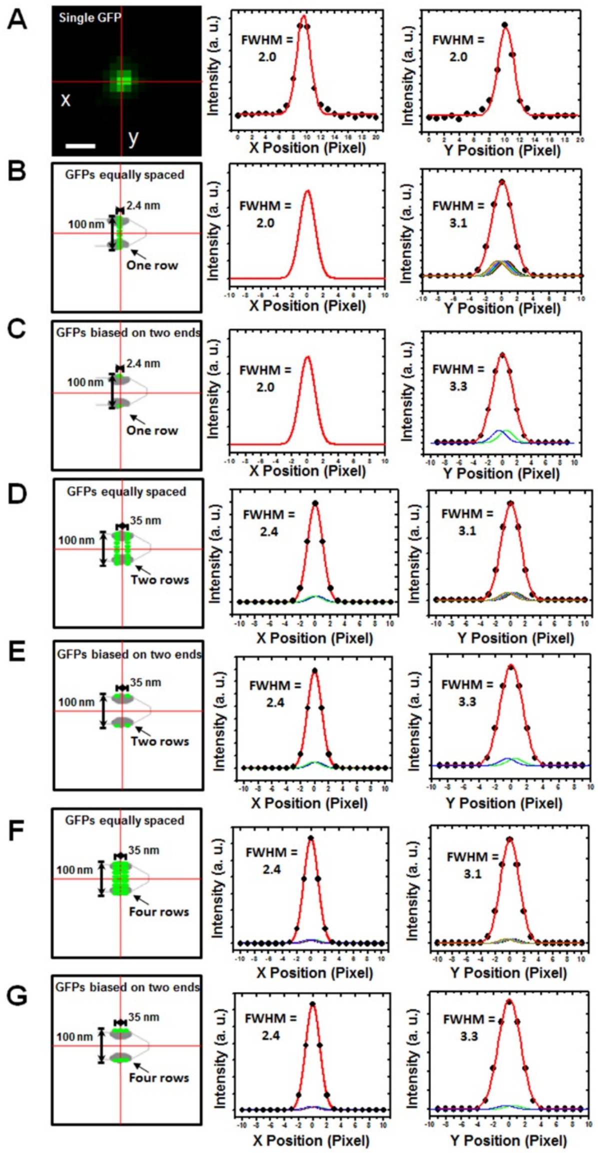

Supplemental Figure S2 | Fitting of a single yeast GFP-NPC by Gaussian functions.

(A) Fluorescent spot of a single stationary GFP molecule detected by SPEED microscopy[1]. The GFP spot is fitted well by a 2D symmetrical Gaussian function with a full width at half maximum (FWHM) of two pixels (240 nm per pixel) in both x and y directions. (B-C) One possible distribution of Nup-GFP (green spots) with 8, 16 or 32 copies aligned in a single row at the cross-section of the yeast NPC (grey schematic). Because multiple copies of each Nup localize in the NPC, the fluorescent spot of a

single GFP-NPC is an overlap of these GFPs. The multiple copies of a Nup may space equally on the cross-section of the nuclear pore (≈ 100 nm outside diameter for yeast NPCs) (2), or, in the worst case, half of them might show a bias for one side and the other half are on the other side. Both situations can be studied by fitting their fluorescence to a sum of Gaussian functions. We have examined 8, 16 or 32 copies of Nups in the NPC. Independence of the number of copies, the fittings resulted in a FWHM of 2 pixels (480 nm) and 3.1 pixels (740 nm) in the x and y directions when Nups are equally spaced and a FWHM of 2 pixels and 3.3 pixels in the x and y directions when they are biased. (D-E) One possible distribution of Nup-GFP with 8, 16 or 32 copies aligned in two rows with a distance of ≈ 35 nm at the cross-section of the yeast NPC[2]. The Gaussian fittings resulted in a FWHM of 2.4 pixels and 3.1 pixels in the x and y directions when Nups are equally spaced and a FWHM of 2.4 pixels and 3.3 pixels in the x and y directions when they are biased. (F-G) Another possible distribution of Nup-GFP with 8, 16 or 32 copies aligned in four rows within a distance of ≈ 35 nm at the cross-section of the yeast NPC. The Gaussian fittings resulted in a FWHM of 2.4 pixels and 3.1 pixels in the x and y directions when Nups are equally spaced and a FWHM of 2.4 pixels and 3.3 pixels in the x and y directions when they are biased. The maximum ratios of the FWHM in the x and the y directions under the above situations are 1.29 and 1.65, respectively. The ratios enabled us to identify a single GFP-NPC (the ratio of its FWHM in the x and y directions is within the range of 1.29 to 1.65), or multiple overlapped GFP-NPCs (the ratio is > 1.65), or other false ones (the ratio is < 1.29) are excited in the illumination volume of SPEED microscopy.

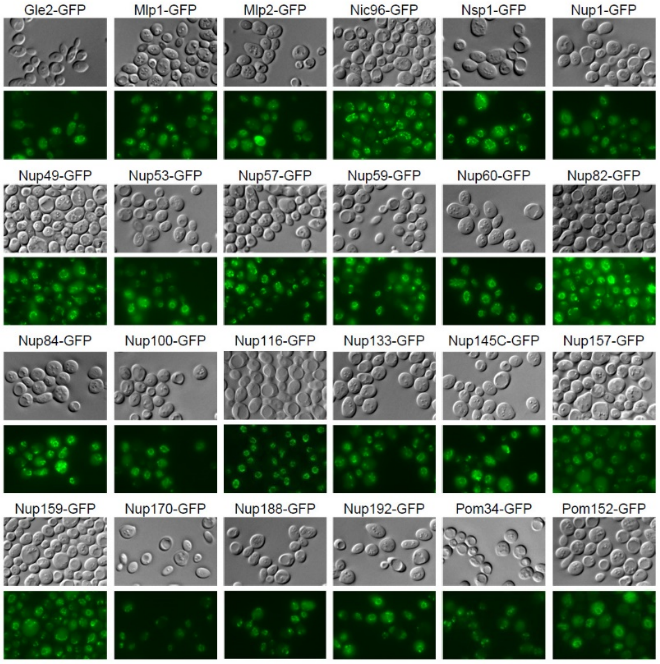

Supplemental Figure S3 | Photographs of yeast expressing different GFP-labeled nucleoporins. These haploid S. cerevisiae strains (except Nup116-GFP) were generated by Huh and coworkers[3] using homologous recombination. Yeast were grown in YPD media at 30°C to a culture density of 0.5-2.0 OD_{600}/mL. They were then harvested by centrifugation, resuspended in minimal media, spotted on a microscope slide, and visualized with a Nikon Eclipse 80i fluorescence microscope using a 100x oil plan fluor objective. The yeast were photographed with a CCD camera with exposure times ranging from 200 to 450 ms. The Nup-GFP chimeras were expressed from their endogenous NUP loci under control of their endogenous NUP promoter. Cells are ~5 µm, and their nuclei ~2 µm.

Suppression of background noise/fluorescence in the measurements of nxGFP molecular complexes *in vitro*

To suppress the background noise/fluorescence, we have taken several major measures in our experiments. First, we kept the concentration of GFP complexes on the coverslip at a single-molecule level to ensure all the single fluorescent spots isolated well spatially (neighboring distance ≥5 μm), as shown in Figure 1. Second, we have washed away the floating fluorescence molecules in our samples after GFP complexes formed and anchored on the coverslip, which significantly reduced the background noise/fluorescence. Third, all the photobleaching curves shown in paper were obtained by subtracting the backgroup fluorescence from the raw data of GFP fluorescence. This subtraction has further ensured that the photobleaching curves we obtained were from the excited GFPs, instead of fluctuations from the camera or the backgroup noise in samples. Finally, SPEED microscopy ensured a diffraction-limited illumination volume in the sample, which also significantly suppressed the background noise and achieved a high (≥11) signal-to-noise (S/N) ratio for single-particle localization.

To resolve steps in photobleaching curves of GFPs *in vitro* and *in vivo*

To identify each photobleaching step, we followed a computer algorithm developed by Watkins & Yang*,* on the basis of the maximum likelihood ratio method.[4] This method allows to quantitatively determine discrete intensity jumps in time-resolved optical single-molecule emission data. There are two major steps in the analyses: First, using a generalized likelihood ratio test to distinguish intensity change points in single-

molecule fluorescence measurements. Basically, both the incoming photons and the dark counts of detectors will be Poisson distributed. Thus, using Poisson function, the probability of containing an intensity change point in the measured data can be calculated and then compared to the probability of data without a changing point. With this comparison, a confidence level and a confidence region for the position of the intensity changing point are determined. Following this procedure, all the number and the location of intensity changes in single-molecule fluorescence curves can be finally identified. Second, clustering the identified change points and avoiding repeatedly counting the same state. Normally, the N change steps represent N+1 different intensity change states in a single-molecule trajectory. Some of these different intensity states have very small difference in intensity and may arise from identical emission level in a molecule. Using an agglomerative hierarchical clustering algorithm and an expectation-maximization procedure,[5] the intensities and probabilities of all possible numbers of states are calculated, which are further compared to the identified states in the first step. Finally, a minimum number of states in the single-molecule fluorescence measurements is decided with the Bayesian Information Criterion.[4] The authors have provided a directly download software package on their lab website for users to apply this method.[4]

1. Ma, J. & Yang, W. Three-dimensional distribution of transient interactions in the nuclear pore complex obtained from single-molecule snapshots. *Proc. Natl. Acad. Sci. USA* **107**, 7305-7310 (2010).

2. Yang, Q., Rout, M. P. & Akey, C. W. Three-dimensional architecture of the Isolated yeast nuclear pore complex: functional and evolutionary implications. *Mol. Cell* **1**, 223-234 (1998).

3. Huh, W. K. *et al.* Global analysis of protein localization in budding yeast. *Nature* **425**, 686-691 (2003).

4. Watkins, L. P. & Yang, H. Detection of Intensity Change Points in Time-Resolved Single-Molecule Measurements. *J. Phys. Chem. B.* 109, 617-628 (2005).

5. Fraley, C. & Raftery, A.E. How many clusters? Which clustering methods? Answers via model-based cluster analysis. *Computer J.*, 41, 578-588 (1998).
